# Optimization of the Composition of Mesoporous Polymer–Ceramic Nanocomposite Granules for Bone Regeneration

**DOI:** 10.3390/molecules28135238

**Published:** 2023-07-06

**Authors:** Marta Trzaskowska, Vladyslav Vivcharenko, Wojciech Franus, Tomasz Goryczka, Adrian Barylski, Agata Przekora

**Affiliations:** 1 Independent Unit of Tissue Engineering and Regenerative Medicine, Medical University of Lublin, Chodzki 1, 20-093 Lublin, Poland; marta.trzaskowska@o2.pl (M.T.); vladyslav.vivcharenko@umlub.pl (V.V.); 2 Department of Construction Materials Engineering and Geoengineering, Lublin University of Technology, Nadbystrzycka 38 D, 20-618 Lublin, Poland; w.franus@pollub.pl; 3 Institute of Materials Engineering, University of Silesia in Katowice, 75 Pułku Piechoty 1A, 41-500 Chorzów, Poland; tomasz.goryczka@us.edu.pl (T.G.); adrian.barylski@us.edu.pl (A.B.)

**Keywords:** biomaterial, bioceramics, calcium phosphates, polymers, biocompatibility, microhardness, cytotoxicity, porosity

## Abstract

Difficult-to-treat bone damage resulting from metabolic bone diseases, mechanical injuries, or tumor resection requires support in the form of biomaterials. The aim of this research was to optimize the concentration of individual components of polymer–ceramic nanocomposite granules (nanofilled polymer composites) for application in orthopedics and maxillofacial surgery to fill small bone defects and stimulate the regeneration process. Two types of granules were made using nanohydroxyapatite (nanoHA) and chitosan-based matrix (agarose/chitosan or curdlan/chitosan), which served as binder for ceramic nanopowder. Different concentrations of the components (nanoHA and curdlan), foaming agent (sodium bicarbonate—NaHCO_3_), and chitosan solvent (acetic acid—CH_3_COOH) were tested during the production process. Agarose and chitosan concentrations were fixed to be 5% *w*/*v* and 2% *w*/*v*, respectively, based on our previous research. Subsequently, the produced granules were subjected to cytotoxicity testing (indirect and direct contact methods), microhardness testing (Young’s modulus evaluation), and microstructure analysis (porosity, specific surface area, and surface roughness) in order to identify the biomaterial with the most favorable properties. The results demonstrated only slight differences among the resultant granules with respect to their microstructural, mechanical, and biological properties. All variants of the biomaterials were non-toxic to a mouse preosteoblast cell line (MC3T3-E1), supported cell growth on their surface, had high porosity (46–51%), and showed relatively high specific surface area (25–33 m^2^/g) and Young’s modulus values (2–10 GPa). Apart from biomaterials containing 8% *w*/*v* curdlan, all samples were predominantly characterized by mesoporosity. Nevertheless, materials with the greatest biomedical potential were obtained using 5% *w*/*v* agarose, 2% *w*/*v* chitosan, and 50% or 70% *w*/*v* nanoHA when the chitosan solvent/foaming agent ratio was equal to 2:2. In the case of the granules containing curdlan/chitosan matrix, the most optimal composition was as follows: 2% *w*/*v* chitosan, 4% *w*/*v* curdlan, and 30% *w*/*v* nanoHA. The obtained test results indicate that both manufactured types of granules are promising implantable biomaterials for filling small bone defects that can be used in maxillofacial surgery.

## 1. Introduction

Bone tissue is the second most frequently transplanted tissue [1]. Difficult-to-treat bone damage, as well as small bone loss, as a result of various diseases or accidents requires support in the form of biomaterials that can restore tissue architecture and function [2]. Artificial biomaterials are subject to the following requirements: biocompatibility, osteoconductivity (capability of the material to ensure the environment for the formation of new bone), bioactivity (a set of material properties enabling the material to connect with the host’s bone tissue), biodegradability, and mechanical strength [2,3]. Among all materials used in bone regenerative medicine, calcium phosphates are the most thoroughly understood group [4]. Belonging to calcium phosphates, hydroxyaptite (HA) most closely resembles the mineral part of the extracellular matrix of bones. Despite favorable features such as high biocompatibility and bioactivity, hydroxyapatite also has significant disadvantages. Poor mechanical properties such as brittleness, high density, and slow biodegradation make it difficult to use in orthopedics and maxillofacial surgery [5,6].

Taking into account the many expectations placed on biomaterials used in tissue engineering, composites that combine various features of at least two types of materials have become the subject of intensive research. Some of the most popular inorganic–organic composite materials consist of plastic and easily degradable polymers, and highly biocompatible calcium phosphate ceramics [7]. In addition, the presence of calcium phosphates improves the bioactivity and osteoconductivity of the implant [5,7]. For example, Lv et al., observed increased proliferation, osteogenic differentiation, and mineral deposition when human mesenchymal stem cells (HMSCs) were cultured on the surface of a scaffold made of poly(lactic-co-glycolic acid) (PLGA) and nanohydroxyapatite (nanoHA) compared with PLGA material [8]. In turn, Tsiourvas et al., noted an improvement in the mechanical properties of composites containing nanoHA and chitosan compared with the chitosan scaffold [9]. Importantly, composite materials are attractive in terms of adapting their properties to the mechanical and physiological requirements of the implantation site due to the possibility of controlling the quantitative contribution and distribution of individual phases [6].

Chitosan is a natural polysaccharide of great interest in regenerative medicine. Particularly due to its biocompatibility and osteoconductive properties, it is often used as a component of bone biomaterials [4]. Furthermore, chitosan as a derivative of the commonly occurring chitin is easily available [10]. Another important feature is biodegradation under physiological conditions, which can be controlled with the molecular weight of the compound or the degree of deacetylation [11]. Infections are a significant problem in orthopedic procedures. Chitosan also exhibits antibacterial activity via several mechanisms, e.g., it causes a change in the permeability of the microbial membrane or reduces the growth of bacteria by binding nutrients [4]. Unlike chitosan, which is soluble in acidic pH solutions, agarose is a polysaccharide that can be dissolved in water or in an alkaline environment [12]. The ability to adjust the mechanical properties and permeability with the appropriate selection of the concentration of self-gelling agarose is a very desirable feature of biomaterial components [12,13]. In addition, agarose is characterized by the lack of cellular toxicity and adverse reactions from the immune system [13,14]. Curdlan is another commonly used polymer that also possesses extraordinary gelling properties. This (1–3)-β-d-glucan is able to form both a poorly hardened gel and a compact gel depending on the applied heating temperature. The use of lower temperatures (55–60 °C) during the heating of the aqueous curdlan suspension results in obtaining a gel with an unstable, reversible structure, while the use of high temperatures (>80 °C) ensures the formation of a stable, compact gel [15,16]. Moreover, curdlan has a number of biological activities valuable in regenerative medicine, such as antibacterial, anticancer, immunomodulatory, and antiviral properties [17,18]. In these studies, based on the experience gained in the production of curdlan-containing biomaterials for bone tissue regeneration, a gelation temperature of 96 °C was used for the production of granules [19,20]. The application of high temperatures ensured the formation of a well-crosslinked, hard, and stable gel with the most optimal mechanical properties for use in the regeneration of bone tissue.

The aim of this research was to optimize the concentration of individual components of nanocomposite granules for the treatment of small bone defects. Two types of biomaterial consisting of calcium phosphate ceramics and polymers were produced: (1) nanoHA/chitosan/agarose granules and (2) nanoHA/chitosan/curdlan granules. It is worth noting that the available scientific literature has never described a material in the form of granules containing curdlan, chitosan, and hydroxyapatite nanopowder. Moreover, the biomaterial production method also has novel aspects. The simultaneous use of a foaming agent (sodium bicarbonate—NaHCO_3_) and a lyophilization process in the production of nancomposite granulates has not been previously described. The application of these two techniques resulted in obtaining granules with high porosity and a relatively high specific surface area (SSA). These parameters are known to have a positive effect on increasing the bioactivity and biodegradation of biomaterials [21]. The method for the production of nanoHA/chitosan/curdlan granules has been claimed at the Polish Patent Office (patent application No. P.442451, 2022). The produced granules were subjected to the evaluation of cytotoxicity, cell growth on the surface of the materials, plane strain Young’s modulus, and microstructure (porosity, average pore size, specific surface area (SSA), surface roughness, SEM) in order to indicate the biomaterials with the most favorable properties.

## 2. Results and Discussion

### 2.1. Determination of Microstructural Properties

In this study, the optimization of the composition of nanocomposite granules for small bone defect treatment was performed. During the production of biomaterials based on agarose, chitosan, and nanohydroxyapatite (nanoHA), various concentrations of the following ingredients were tested:

- Nanohydroxyapatite: 50 and 70% *w*/*v* relative to a suspension of polymers in acetic acid (CH_3_COOH) solution;
- Solvent (CH_3_COOH): 1 and 2% *v*/*v* relative to distilled water;
- Foaming agent (bicarbonate—NaHCO_3_): 1 and 2% *w*/*v* relative to a suspension of polymers in CH_3_COOH solution.

Concentrations of the solvent and foaming agent were applied at two different ratios, 1:1 and 2:2, which were previously selected as the most optimal [22]. Agarose and chitosan concentrations were fixed to be 5% *w*/*v* and 2% *w*/*v* relative to a solution of CH_3_COOH, respectively, based on our previous research. Since the production process of nanoHA/chitosan/agarose granules demonstrated that the best microstructure is obtained when 2% *v*/*v* solvent (CH_3_COOH) and 2% *w*/*v* foaming agent (NaHCO_3_) are applied, nanoHA/chitosan/curdlan nanocomposite was produced using the 2:2 ratio of the acetic acid and sodium bicarbonate. Thus, during the formulation of materials made of chitosan, curdlan, and nanoHA, different concentrations of the following components were used:

- Nanohydroxyatatite: 30 and 50% *w*/*v* relative to a suspension of polymers in CH_3_COOH solution;
- Curdlan: 4 and 8% *w*/*v* relative to a solution of CH_3_COOH.

**Figure 1:**
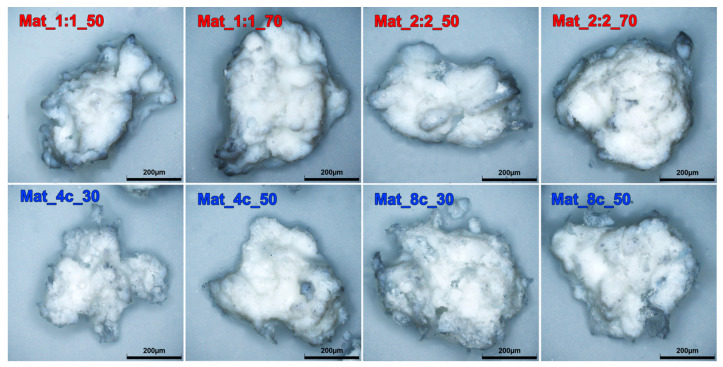
Stereoscopic images of produced granules (red color font represents agarose-based granules, while blue color font represents curdlan-based samples).

It should be noted that the optimization of the concentrations of individual components is very important, since the selected ingredients may affect the essential properties of the granules. The content of nanohydroxyapatite in a biomaterial affects the adhesion, proliferation, and differentiation of bone cells and is crucial to the microstructure and mechanical properties of the biomaterial [21]. The CH_3_COOH-to-NaHCO_3_ ratio influences the porosity and specific surface area (SSA) of granules. In turn, the presence of curdlan, which is a binder for nanoHA, is of great importance for the stability and mechanical parameters of the resultant biomaterial. Table 1 presents the composition of each variant of the produced biomaterial along with its designation. The stereoscopic images of the produced granule variants are shown in Figure 1.

The pores present in a biomaterial perform many important functions. Above all, they create space for the proliferation and migration of osteoblasts and mesenchymal stem cells and enable the formation of new blood vessels, thus contributing to the regeneration of bone tissue [23]. High porosity is associated with an increase in SSA, which provides an acceleration of protein adsorption, ion exchange with the environment, and bioactivity [24].

All produced types of granulates had a relatively high SSA in the range of 23.0–32.8 m^2^/g and high porosity in the range of 46.4–51.1% (Table 2). The porosity of the produced composite materials was within the range of porosity present in human spongy bone (30–90%) [25]. The increase in the content of ceramics and in the concentration of CH_3_COOH and NaHCO_3_ in the materials with chitosan and agarose did not affect the percentage of porosity of the granulates. In the case of materials with chitosan and curdlan, a slight decrease in porosity was observed with an increase in the content of nanoHA and curdlan; however, the observed differences among the samples were not statistically significant. In the case of SSA, statistically significant differences were observed among the granules produced with the same ingredients applied at different concentrations. The increase in the content of nanoHA in biomaterials with chitosan and agarose resulted in a different effect depending on the concentrations of NaHCO_3_ and CH_3_COOH used. Lower concentrations of these two compounds (1:1 ratio) resulted in a decrease in SSA with an increase in nanoHA content, while higher concentrations of NaHCO_3_ and CH_3_COOH (2:2 ratio) resulted in the opposite trend. This is consistent with the results obtained for biomaterials with chitosan and curdlan, in the production of which higher concentrations of NaHCO_3_ and CH_3_COOH were used. Thus, in the case of granules based on curdlan/chitosan matrix, the value of the SSA increased with the content of nanoHA.

**Figure 2:**
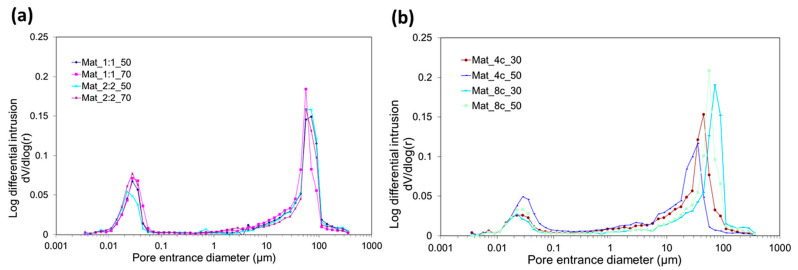
Pore size distribution of biomaterials, (**a**) nanoHA/chitosan/agarose granules and (**b**) nanoHA/chitosan/curdlan granules, determined with the MIP method.

The aim of this study was to produce mesoporous granules. According to the available literature, mesoporous biomaterials show a pore size in the range of 2–50 μm. Pores <2 µm are typical of microporosity, whereas pores > 50 µm indicate macroporosity [26]. The performed analysis of pore size distribution revealed that most of the produced granules were characterized by the highest amount of mesopores. It was observed that the samples with agarose and chitosan contained a large number of micropores (0.02–0.03 µm), but the dominant type of pores was mesopores in the range of 40–50 µm with some macropores in the range of 60–90 µm (Figure 2a). Biomaterials made of curdlan/chitosan matrix contained some micropores in their structure and, depending on the curdlan content, a great number of mesopores or macropores. The samples with 4% *w*/*v* curdlan were primarily characterized by mesoporosity (20–50 µm), while the granules with 8% *w*/*v* curdlan predominantly contained macropores (60–100 µm) (Figure 2b). Importantly, for all samples, apart from the those containing 8% *w*/*v* curdlan, the calculated average pore size (which was in the range of 2.5–7.2 μm) reflected the mesoporosity trend.

In all tested types of granulates, the average pore diameter decreased with the increase in nanoHA content. The observed dependence between HA content and pore diameter was most likely related to the varying density of the raw paste material. The introduction of pores in the biomaterial microstructure using the gas foaming method (reaction of acetic acid with NaHCO_3_) was hindered in the case of denser paste having higher content of nanoHA. Although the size of micropores and mesopores is too small to provide space for blood vessel ingrowth, they can effectively increase the SSA, thereby stimulating bioactivity and osteogenesis with increased ion exchange with the environment [27]. Studies also showed that biomaterials with a mesoporous structure can be effective carriers for the delivery of growth factors over a long period of time [28]. Moreover, although it is commonly known that macropores (>50 μm) are the most desired to facilitate bone ingrowth deep into implants [26], some scientists have proven that mesoporosity also favors osteoblast adhesion and proliferation. Akin et al. noted an increase in the proliferation of human bone-derived cells (HBDCs) on the surface of TiO_2_ film covering a titanium material with smaller pores, i.e., 0.50 and 16 μm compared with 50 μm pores [29]. In another study, it was reported that HA/silica sol/sodium tripolyphosphate composite material with pores of 5–25 µm promoted the adhesion and growth of osteoblast-like cells [28].

**Figure 3:**
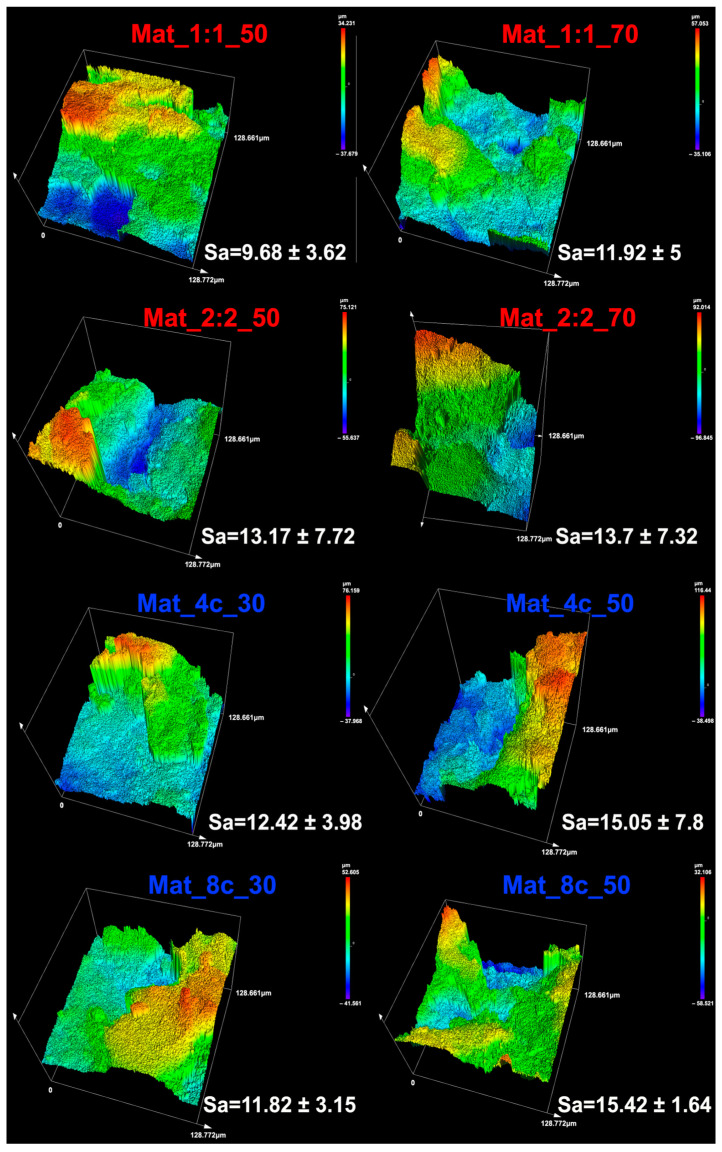
Topography of granule surface and areal surface roughness (S_a_) measured with confocal laser scanning optical profilometry (red color font represents agarose-based granules, while blue color font represents curdlan-based samples).

Surface roughness is an important parameter of surface topography and is expressed by measuring depressions and elevations on the surface of a material. Modification of the surface texture may affect the type of cells that adhere to it; for instance, a rough surface promotes the adhesion of osteoblasts, while a smoother surface is favored by periodontal fibroblasts or epithelial cells [30]. The results obtained with confocal laser scanning optical profilometry show that all types of produced granules were characterized by high areal surface roughness (S_a_) in the range of 9.69–15.42 µm. Biomaterials with chitosan/agarose matrix had less uniform surface topography than granules with chitosan/curdlan matrix, which is reflected in the values of the standard deviations of the calculated S_a_ values. In the case of biomaterials with chitosan/curdlan matrix, a slight increase in areal surface roughness was observed with the increase in the content of nanoHA in the samples; nevertheless, no statistically significant differences were detected (Figure 3).

**Figure 4:**
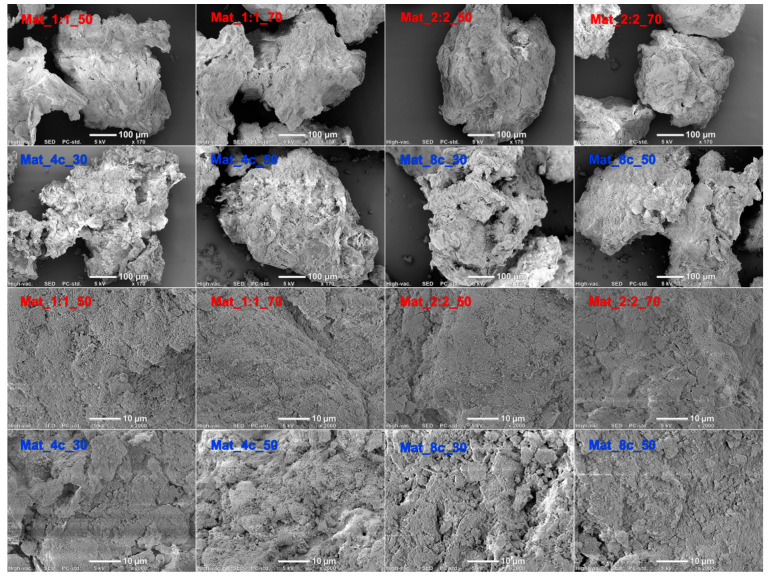
SEM images of the produced nanocomposite granules (red color font represents agarose-based granules, while blue color font represents curdlan-based samples).

SEM analysis did not show noticeable differences in the morphology or surface microstructure of granules produced with the same ingredients. However, SEM images revealed that the granules with curdlan/chitosan matrix were characterized by more irregular shape, sharp edges, and rougher surface with numerous cracks compared with the granules with agarose/chitosan matrix, which had a more compact and denser microstructure (Figure 4).

### 2.2. Microhardness Testing

**Figure 5:**
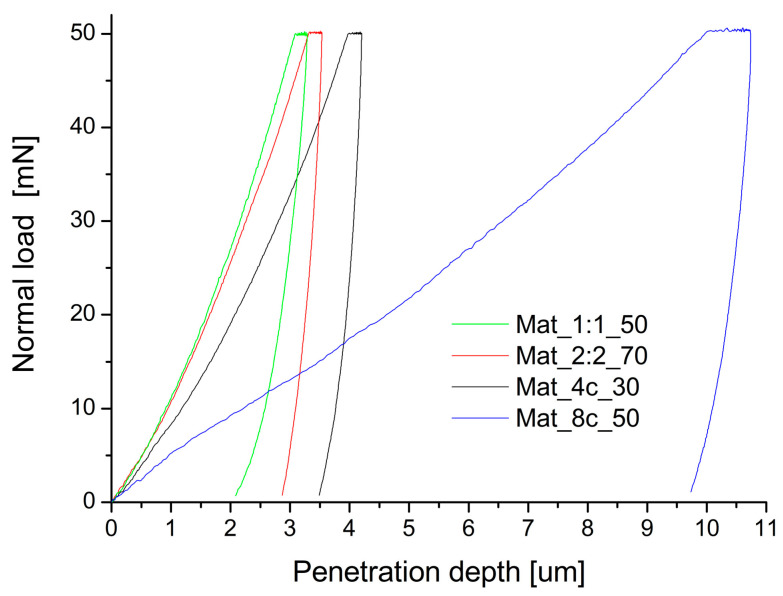
Exemplary dependence of indentation force on indentation depth measured in the microhardness test.

The biomaterial should be characterized by mechanical parameters similar to those of bone tissue, ensuring the desired strength and rigidity. It has been shown that cell behavior can be regulated by surface morphology and stiffness. Young’s modulus, measured as the resistance of a material to tensile or compressive force, is often used to determine tissue stiffness [31]. In order to determine Young’s modulus, indentation curves were measured using microhardness testing (the relationship between normal load and penetration depth). Exemplary results are shown in Figure 5.

**Figure 6:**
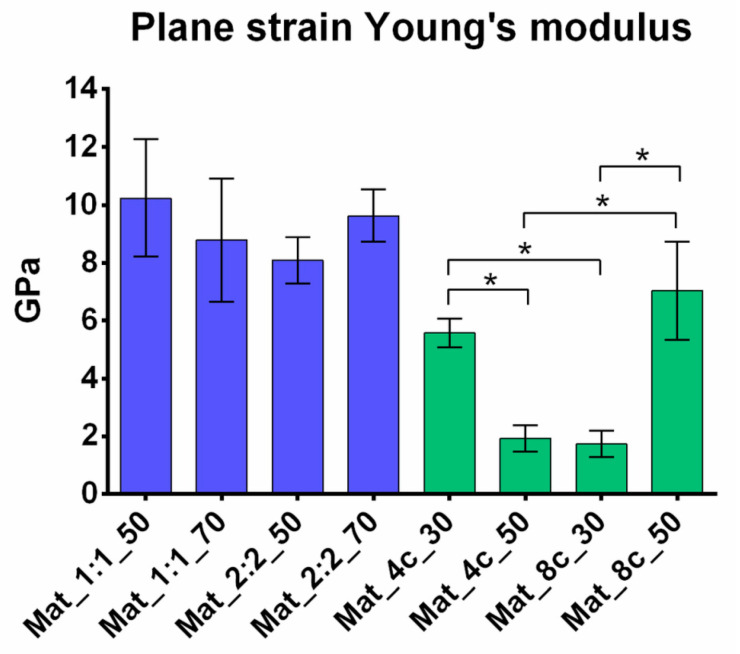
Plane strain Young’s modulus of the granules obtained with microhardness testing (* statistically significant results with *p*-value < 0.05; one-way ANOVA followed by Tukey’s test).

The determined values show that the manufactured variants of granules were characterized by plane strain elasticity in the range of 1.73–10.24 GPa (Figure 6).

The Young’s modulus of the human trabecular bone given in the literature may vary depending on the measurement method used, the patient’s condition, sex, age, or anatomical location of the bone fragment [32]. For example, using the nanoindentation method, the Young’s modulus of the human trabecular bone was between 1.28 and 22.34 GPa [32]. This range includes the values obtained for the tested materials with microhardness measurement. Granules consisting of agarose, chitosan, and nanoHA had lower elasticity (8.09–10.24 GPa) than materials with curdlan, chitosan, and nanoHA (1.73–7.04 GPa). Importantly, the Young’s modulus values of the different variants of nanoHA/chitosan/agarose granules did not significantly vary. In the case of granules made of chitosan/curdlan matrix, the Mat_4c_30 and Mat_8c_50 samples revealed significantly higher Young’s modulus values than other nanoHA/chitosan/curdlan variants. It was assumed that the higher Young’s modulus of Mat_8c_50 primarily resulted from higher content of nanoHA (50%). In the case of Mat_4c_30, higher stiffness was most likely a result of the specific production process. Before granulation, the Mat_4c_30 sample was made using nanoHA powder covered by very soft polymer. Importantly, the small content of nanoHA (30%) made it difficult to obtain its uniform distribution in raw paste before the thermal gelation process. Thus, nanoHA formed aggregates. During the granulation process of the Mat_4c_30 sample, soft polymer matrix chipped off from the ceramic phase, leaving aggregates of nanoHA. Thus, the resultant biomaterial had in fact low content of polymer matrix and showed higher stiffness than Mat_4c_50, which had higher content of nanoHA and in which the polymer did not extensively chip off during granulation. In turn, Mat_8c_30 had higher content of curdlan (8%) than Mat_4c_30; thus, its polymer matrix was more stable (thermally gelled 8% curdlan is denser) and did not crumble during the granulation process, making the material more elastic than Mat_4c_30. Therefore, specific steps of the production process could have significantly changed the final content of the individual components of the granules, affecting their microstructural and mechanical properties. For this reason, the evaluation of the content of polymer and ceramic phases in the final product after the granulation process is necessary and is going to be carried out in our future studies. 

There are only a few composite materials in the form of granules described in the literature. Zima et al. investigated the mechanical properties of hybrid hydroxyapatite and chitosan granules, as well as hydroxyapatite granules. It was noted that the addition of 17% and 23% chitosan to HA granules resulted in 12-fold and 16-fold increases in compressive strength, respectively, compared with pure HA granules. The authors suggested that good adhesion and chemical interactions among the material components were responsible for this phenomenon [33]. Compressive strength and Young’s modulus are directly proportional to each other [34]. Lee et al. demonstrated that the Young’s modulus values of porous granules with β-tricalcium phosphate were 0.24, 0.59, and 0.37 GPa [35]. Most of the reports present in the literature concern composite materials in which only the ceramic phase is in the form of granules and is surrounded by polymer matrix [36,37]. In turn, bone scaffold made of chitosan, β-1,3-glucan (curdlan), and hydroxyapatite granules showed high flexibility, as its Young’s modulus was only 0.25 ± 0.03 MPa [38]. The Young’s modulus values determined for the 3D printed scaffold made of polycaprolactone (PCL) and biphasic calcium phosphate (BCP) microparticles were 44.86 MPa for the solid form and 8.00 MPa for the porous form [39].

### 2.3. Cytotoxicity Tests

**Figure 7:**
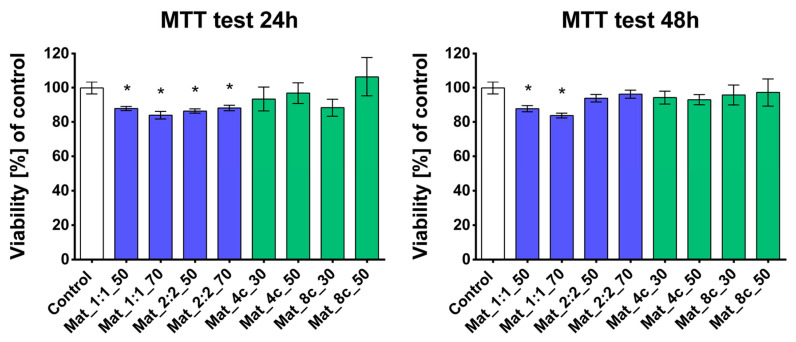
Indirect cytotoxicity test based on MTT assay conducted after 24 and 48 h according to ISO 10993-5 [42] (* statistically significant results compared with control cells with *p*-value < 0.05; one-way ANOVA followed by Tukey’s test).

Biocompatibility is the fundamental required property of a biomaterial intended for implantation. A biocompatible material has no negative influence on the physiological activity of cells and does not interfere with the normal functioning of the host organism. It is associated with a lack of immunogenicity, mutagenicity, genotoxicity, and cytotoxicity [40,41]. As a result of optimizing biomaterial production, variants of granules differing in the composition of individual components were formed. The cytotoxicity of the biomaterials was assessed with the MTT assay and live/dead fluorescent staining. All tested materials were non-toxic to the MC3TC-E1 cell line after exposure to biomaterial extracts at two time points. Cell viability after 24 and 48 h of incubation with each extract of the tested granules exceeded 83% (Figure 7).

**Figure 8:**
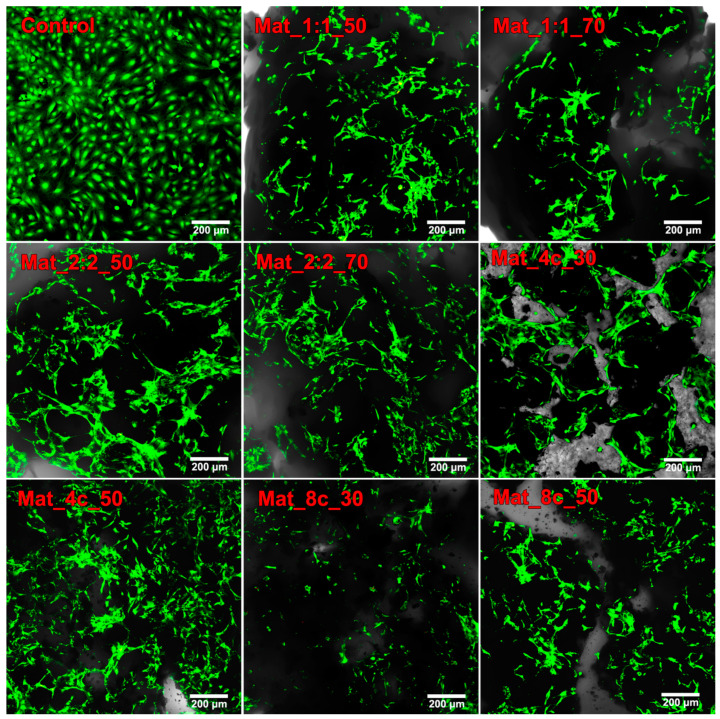
Live/dead staining of BJ MC3T3-E1 cells cultured for 72 h on the biomaterials and polystyrene (green fluorescent was emitted by viable cells, and red fluorescent was emitted by dead preosteoblasts; magnification 100×; scale bar = 200 µm).

According to the ISO 10993-5 (2009) [42] standard, a material can be considered cytotoxic if cell viability after contact with the biomaterial extract is below 70% compared with control cells. Interestingly, the cells showed higher viability after exposure to extracts of biomaterials containing curdlan compared with agarose. The cytotoxicity test in direct contact with the biomaterials revealed that all tested granulates favored cell adhesion. The surface of the biomaterials was covered with viable preosteoblasts at high density (cells emitting green fluorescence) (Figure 8). However, a noticeably lower number of cells were observed on the biomaterial surface containing 8% *w*/*v* curdlan and 30% *w*/*v* nanoHA. The obtained result can be explained by the worse osteoconductivity of Mat_8c_30 granules resulting from a lower concentration of the nanoHA compound in the sample [5,43]. Interestingly, sample Mat_4c_30, which was made of 30% *w*/*v* nanoHA and 4% *w*/*v* curdlan, supported cell adhesion and growth. This was most likely caused by the higher stiffness of this sample, which resulted from the specific production process, as explained above (Section 2.2) The obtained result of no cytotoxicity is consistent with the available scientific literature where scientists assessed the cytotoxicity of biomaterials of similar composition. Przekora et al. investigated a scaffold consisting of chitosan, β-1,3-glucan (curdlan), and commercially available hydroxyapatite granules (HA BIOCER; Chema-Elektromet Spoldzielnia Pracy, Poland). Using the WST-8 and NRU cytotoxicity tests, as well as observation of the cells after live/dead double staining, it was shown that the material did not reduce the viability of human fetal osteoblastic cells (hFOBs) and favored their adhesion to the surface of the biomaterial [38]. Kazimierczak et al. demonstrated that scaffold containing chitosan, agarose, and nanoHA powder was non-toxic to MC3T3-E1 and hFOB 1.19 cells. Cell viability after incubation with the material extract after 24 h was close to 100%, and after 48 h, it decreased by about 10–20% [26].

## 3. Materials and Methods

### 3.1. Production of Granules

To produce nanocomposite granules, chitosan (Sigma-Aldrich Chemicals, Warsaw, Poland) was dissolved in acetic acid solution (Avantor Performance Materials, Gliwice, Poland). Then, the following components were added to the obtained chitosan solution: agarose (purchased from Sigma-Aldrich Chemicals, Warsaw, Poland), in the case of nanoHA/chitosan/agarose granules, or curdlan (purchased from Wako pure Chemicals Industries, Japan), in the case of nanoHA/chitosan/curdlan granules; nanohydroxyapatite powder (Sigma-Aldrich Chemicals, Warsaw, Poland); and sodium bicarbonate (Sigma-Aldrich Chemicals, Warsaw, Poland). According to the manufacturer’s specification sheet, nanoHA was characterized by the following parameters: particle size ≤ 200 nm (BET) and specific surface area ≥ 9.4 m^2^/g. The phase composition of the nanopowder was confirmed in this study using the X-ray diffraction (XRD) method. The diffractogram only showed reflections originating from hydroxyapatite, which was identified according to the strongest and characteristic interplanar distances d*_hkl_* = 2.814, 2,783, 2,718, 3,449, 1.845, and 1.946 Å. The obtained blend was mixed thoroughly. The finished paste was placed in a mold and incubated in a water bath at 95 °C for 20 min. After this time, the materials were cooled, frozen at −80 °C, and then subjected to the lyophilization process. The resulting materials were soaked in PBS solution for 1.5 h and dried for 24 h at room temperature. The last step was granulation in a mortar and collection of the granules using sieves to obtain the grain size of 0.3–0.4 mm. The materials were sterilized using ethylene oxide.

### 3.2. Determination of Porosity and Specific Surface Area

The degree of porosity (area occupied by pores) and the average pore size were assessed using the mercury intrusion porosimetry method. The dried samples were placed in measuring vessels and degassed. Then, the samples were immersed in mercury, which was forced into the pores using external pressure. As the pressure increased, the size of the pores into which mercury could penetrate decreased. The measurement of the specific surface area of the granulates was carried out using the nitrogen adsorption technique according to the Brunauer–Emmett–Teller theory. The measurements of porosity and SSA were conducted 3 times on 3 independent samples (n = 3).

### 3.3. Determination of Surface Topography

The surface roughness of the granules was measured using a confocal laser scanning optical profilometer (LEXT™ OLS5100 3D Scanning Laser Microscope; Olympus Corporation, Tokyo, Japan). Average areal surface roughness (S_a_) measurements were performed on the surface of 10 different granules at 2101× magnification. The scanning area was 128.772 μm × 128.661 μm. To visualize the microstructure of the entire granules, images of biomaterials were taken using a stereoscopic microscope (Olympus SZ61TR; Olympus Polska Sp. z o. o., Warsaw, Poland). In addition, the topography and surface microstructure of biomaterials were determined using a scanning electron microscope (SEM; FEI Nova NanoSem 450; Tokyo, Japan). Before imaging, the samples were coated with a thin gold layer of 15 nm. The images were obtained at the accelerating voltage of 5 kV.

### 3.4. Microhardness Testing

The plane strain modulus of elasticity of the granules was tested using a Micro Combi Tester—MCT3 microhardness tester (Anton Paar, Corcelles-Cormondrèche, Switzerland). The procedure was carried out in accordance with the ISO 14577 standard [44]. Before the measurement, the granules were embedded in resin and polished. A Berkovich diamond indenter (SN: B-V83; α = 65.3° +/− 0.3°) with a maximum load of 50 mN was used for the measurement. The loading and unloading of the indenter were performed at the speed of 100 mN/min. The maximum load time was 10 s, and 10 indention prints were made on each sample. Microhardness HIT and Young’s modulus EIT were determined using the standard Oliver–Pharr method [45], which uses the slope tangent to the initial part of the unloading curve in the calculations.

### 3.5. In Vitro Cell Culture Experiments

In vitro cytotoxicity experiments were performed using a mouse calvarial preosteoblast cell line (MC3T3-E1 Subclone 4; ATCC-LGC, standards, Teddington, UK). Cells were cultured in alpha MEM medium (Gibco, Life technologies, Carlsbad, CA, USA) supplemented with 10% fetal bovine serum (FBS; Pan-Biotech GmbH, Aidenbach, Bavaria, Germany) and the following antibiotics: 100 U/mL penicillin (Sigma-Aldrich Chemicals, Warsaw, Poland) and 0.1 mg/mL streptomycin (Sigma-Aldrich Chemicals, Warsaw, Poland). MC3T3-E1 cells were incubated at 37 °C with 5% CO_2_ in air atmosphere.

#### 3.5.1. Indirect Cytotoxicity Test

The cytotoxicity of the fabricated materials was evaluated using the procedure described by ISO 10993-5 (2009) [42]. The viability of MC3T3-E1 cells in indirect contact with the samples was measured after exposing the cells to liquid extracts of the biomaterials. Cells were seeded into a 96-well plate in culture medium at a density of 2 × 10^4^ cells per well and cultured for 24 h. Simultaneously, extracts were prepared by incubating the granules in culture medium at 37 °C for 24 h (100 mg of material per 1 mL of medium). Medium incubated in a well of a polystyrene plate without tested granules was used as control. After 24 h, the cell medium was discarded, and the appropriate biomaterial extract or control medium was added to the wells; then, the cells were incubated for another 24 or 48 h. Cell viability was assessed with the MTT colorimetric assay (Sigma-Aldrich Chemical, Warsaw, Poland) according to a previously described procedure [46]. The results were presented as percentages of the absorbance value of the control. The test was carried out in 3 independent repetitions.

#### 3.5.2. Direct Cytotoxicity Test

The cytotoxic effect of the biomaterials on cells was also evaluated in direct contact with the granules. For this purpose, the samples were placed in the wells of a 24-well plate and soaked for one hour in complete culture medium. Then, cells were seeded directly on the granules at a density of 2 × 10^5^ in 500 µL of culture medium and incubated for 72 h at 37 °C. Control cells were seeded on the bottom of a polystyrene well. During the cytotoxicity assessment, cells were fluorescently stained using a Live/Dead Double Staining Kit (Sigma-Aldrich Chemical, Warsaw, Poland) according to the manufacturer’s instructions. Stained cells were observed using a confocal laser scanning microscope (CLSM; Olympus Fluoview equipped with FV1000; Olympus Polska Sp. z o. o., Warsaw, Poland).

## 4. Conclusions

Biomaterials in the form of granules are mainly used during dental procedures and in maxillofacial surgery, e.g., for filling the alveolar ridge and for periodontal regeneration [47,48]. The research results reveal that the granules obtained from nanohydroxyapatite and biopolymer matrix, chitosan/agarose or chitosan/curdlan, were biocompatible and had sufficient microstructural and mechanical properties. The porosity and Young’s modulus of the materials were close to the values characteristic of human trabecular bone. According to the ISO 10993-5 (2009) [42] standard, none of the produced biomaterials was cytotoxic to mouse preosteoblasts. In addition, all produced granules allowed the adhesion and growth of MC3T3-E1 cells on their surface. Taking into account the results of the cytotoxicity studies, and mechanical and microstructural parameters, granules with the greatest biomedical potential were obtained using 5% *w*/*v* agarose, 2% *w*/*v* chitosan, and 50% or 70% *w*/*v* nanoHA when the chitosan solvent/foaming agent ratio was equal to 2:2. In the case of granules containing curdlan/chitosan matrix, the most optimal composition was as follows: 2% *w*/*v* chitosan, 4% *w*/*v* curdlan, and 30% *w*/*v* nanoHA. The above-listed variants of the granules were characterized by good biocompatibility, high porosity with predominantly mesopores, and relatively high SSA and Young’s modulus values. It is also worth noting that in the current literature, there is no described biomaterial in the form of granules used for the regeneration of bone tissue consisting of curdlan, chitosan, and nanohydroxyapatite. The presented specification of granulates indicates that they are good candidates to be used as implantable materials for filling small bone defects. Nevertheless, in order to confirm their high biomedical potential in clinical applications, further detailed research is needed to extensively assess the chemical, physical, and biological properties of the nanocomposite granulates.

## 5. Patents

The method for the production of nanoHA/chitosan/curdlan granules has been claimed at the Polish Patent Office (patent application No. P.442451, 2022).

## Figures and Tables

**Table 1:** Composition of the granules and their designations that were used throughout the manuscript.

Sample	CH_3_COOH (% *v*/*v*)	NaHCO_3_ (% *w*/*v*)	nanoHA (% *w*/*v*)	Chitosan (% *w*/*v*)	Agarose (% *w*/*v*)	Curdlan (s% *w*/*v*)
Mat_1:1_50	1	1	50	2	5	-
Mat_1:1_70	1	1	70	2	5	-
Mat_2:2_50	2	2	50	2	5	-
Mat_2:2_70	2	2	70	2	5	-
Mat_4c_30	2	2	30	2	-	4
Mat_4c_50	2	2	50	2	-	4
Mat_8c_30	2	2	30	2	-	8
Mat_8c_50	2	2	50	2	-	8

**Table 2:** Microstructural characteristics of the granules (porosity and average pore diameter were measured using mercury intrusion porosimetry, whereas SSA was determined using the nitrogen adsorption technique according to the Brunauer–Emmett–Teller theory).

Material	Porosity (%)	Average Pore Diameter (µm)	SSA (m^2^/g)
Mat_1:1_50	47.4 ± 1.2	6.4	32.8 ± 0.4 ^b,c^
Mat_1:1_70	47.0 ± 1.7	4.2	28.7 ± 0.2 ^a,c,d^
Mat_2:2_50	46.9 ± 1.9	7.2	30.2 ± 0.3 ^a,b,d^
Mat_2:2_70	46.4 ± 0.6	3.8	32.3 ± 0.2 ^b,c^
Mat_4c_30	51.1 ± 0.9	6.4	24.8 ± 0.2 ^f,g,h^
Mat_4c_50	47.3 ± 1.4	2.5	29.7 ± 0.1 ^e,g^
Mat_8c_30	49.3 ± 3.3	12.9	23.0 ± 0.1 ^e,f,h^
Mat_8c_50	47.0 ± 2.3	9.0	29.5 ± 0.1 ^e,g^

Statistically significant results compared with ^a^ Mat_1:1_50, ^b^ Mat_1:1_70, ^c^ Mat_2:2_50, ^d^ Mat_2:2_70, ^e^ Mat_4c_30, ^f^ Mat_4c_50, ^g^ Mat_8c_30, and ^h^ Mat_8c_50, where *p*-value < 0.05; one-way ANOVA followed by Tukey’s test; n = 3.

## Data Availability

The datasets used and/or analyzed during the current study are available from the corresponding author (agata.przekora@umlub.pl) on reasonable request.

## Sample Availability

Samples are not available from the authors.
